# Surgical technique for laparoscopic removal of bulky para-aortic nodes without repositioning surgical field during laparoscopic debulking for advanced ovarian cancer

**DOI:** 10.52054/FVVO.14.2.029

**Published:** 2022-07-01

**Authors:** A Puppo, E Olearo, M Ceccaroni

**Affiliations:** Department of Obstetrics and Gynecology, Azienda Ospedaliera Santa Croce e Carle, Cuneo, Italy; Department of Obstetrics and Gynecology, Gynecologic Oncology, and Minimally Invasive Pelvic Surgery, International School of Surgical Anatomy, Istituto di Ricovero e Cura a Carattere Scientifico IRCCS Sacro Cuore-Don Calabria Hospital, Negrar, Verona, Italy

**Keywords:** ovarian cancer, laparoscopy, para-aortic nodes

## Abstract

**Background:**

In the last years, laparoscopy has been progressively introduced in the management of advanced- stage ovarian cancer (AOC) not only to evaluate tumour resectability, but also to perform primary or interval minimally invasive debulking surgery in selected patients. During laparoscopic debulking for AOC, the need to change the surgical field to treat disease in the upper abdomen can be a time-consuming procedure.

**Objective:**

To demonstrate feasibility, safety and effectiveness of laparoscopic approach to remove bulky para-aortic nodes in AOC with a 30-degree 3D-endoscope without repositioning the laparoscopic surgical field.

**Materials and Methods:**

A 51-year-old woman was referred to our centre due to AOC with bulky para-aortic nodes (7 cm polylobate mass at CT-scan). The narrated surgical video article demonstrates the surgical steps for laparoscopic removal of bulky para-aortic nodes with a 30-degree 3D-endoscope, maintaining the vision from the upper abdomen perpendicular to the main axis of the vascular structures for the whole duration of the surgery (“top-bottom” view), without repositioning surgical field.

**Main outcomes measured:**

Complete laparoscopic excision of disease was achieved.

**Results:**

Post-operative course was uneventful. Patient recovered from surgery and was able to start adjuvant chemotherapy within 30 days from surgery.

**Conclusions:**

Repositioning the surgical field to perform para-aortic dissection can be a time-consuming procedure during laparoscopic debulking for ovarian cancer. Laparoscopic removal of bulky para-aortic nodes with a 30-degree 3D-endoscope and “top-bottom view” is feasible, safe and effective

## Learning objective

Setting the operating room; setting the laparoscopic surgical field; knowledge of surgical anatomy of the retroperitoneum; surgical steps for safe and complete laparoscopic removal of metastatic para- aortic nodes.

## Introduction

The traditional approach to the treatment of advanced ovarian cancer (AOC) includes laparotomic midline incision to expose the peritoneal cavity (Querleu et al., 2017). In recent years, minimally invasive surgical techniques have increasingly been used in gynaecologic oncology practice because they offer multiple advantages over traditional laparotomy such as smaller incisions, improved visualisation, less blood loss, reduction of need for analgesics, decreased morbidity, more rapid recovery and shorter interval to the initiation of adjuvant therapy. The main concern related to the use of minimally invasive surgery in AOC is the risk of not achieving optimal cytoreduction. However, the technical and clinical feasibility of laparoscopic cytoreduction in selected ovarian cancer patients with limited carcinomatosis or lymph node involvement has already been demonstrated (Gallotta et al., 2016). Laparoscopic debulking can be a long procedure, requiring significant effort from the surgical team: the need to change surgical field in order to access the abdomen in addition to the pelvis can be a time-consuming procedure. Therefore, we would like to demonstrate the feasibility, safety and effectiveness of the laparoscopic approach to remove bulky para-aortic nodes in AOC with a 30-degree 3D-endoscope, without repositioning the laparoscopic surgical field.

## Patient and method

A 51-year-old woman was referred to our centre with a swollen abdomen and pelvic pain. The MRI revealed a large adnexal mass of 20cm and the CT scan confirmed the ovarian mass and bulky para- aortic nodes described as a 7cm polylobate mass at the level of descending aorta and vena cava, just caudad to the left renal vein. Ca 125 was 3577 U/ mL.

She was scheduled for laparoscopic removal of the pelvic mass and mini-laparotomy to extract the specimen: the intra-operative pathology analysis confirmed high grade serous ovarian cancer, therefore, since the Fagotti-score (Fagotti et al., 2006) was less than 8, laparoscopic upfront surgery for cytoreduction was undertaken, including removal of bulky para-aortic nodes.

**Figure 1:**
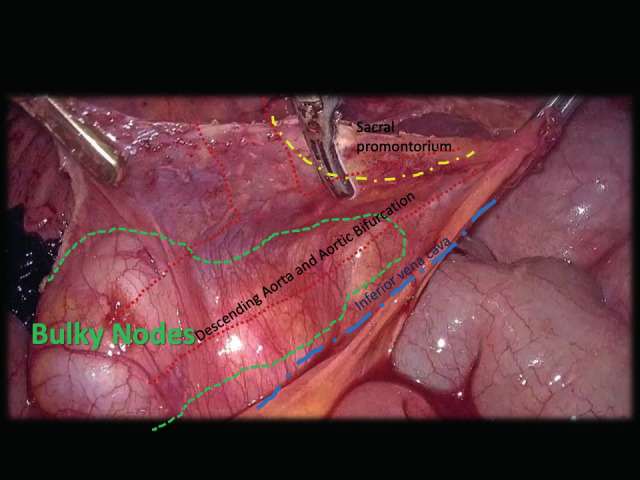


Intervention: After positioning the patient on the surgical table (lithotomy position, 30° Trendelenburg), a 30-degree 3D-endoscope was inserted into the peritoneal cavity in a 12mm access 3cm above the umbilical scar, plus three 5mm accesses in the lower pelvis. The bulky nodes were packed in a 7cm mass adherent to the main vessels, up to the level of left renal vein (Figure 1). Laparoscopic primary cytoreductive surgery was performed, maintaining the vision from the upper abdomen perpendicular to the main axis of the vascular structures for the whole duration of the surgery (“top-bottom” view), without repositioning the surgical field.

The removal of the bulky nodes required opening the retroperitoneum, exposing the psoas muscles, ureters and major vessels (inferior vena cava, inferior mesenteric artery, descending aorta, ovarian vessels up to the level of the left renal vein), progressive dissection and removal of the bulky para-aortic nodes.

## Results

Complete laparoscopic excision of disease was achieved. The final pathology report confirmed high-grade serous ovarian cancer, FIGO Stage IIIC.

The post-operative course was uneventful; the patient was discharged home on day 4 after surgery. She recovered and was able to start adjuvant chemotherapy within 30 days after surgery.

## Discussion

Despite concerns regarding the ability to explore the full extent of the peritoneal cavity and achieve optimal cytoreduction, laparoscopic surgery with its magnification of tissue and increased angle of view can offer to skilled surgeons the best access to inspect and treat metastatic disease in the upper abdomen in selected cases of AOC. In fact, a recent review of 17 studies (Gueli Alletti et al., 2019), considering the role of laparoscopy in primary and interval surgery in AOC, reported an overlap of oncological outcomes compared to traditional surgery. Evidence of benefits about peri and post- operative morbidities, without compromising oncological outcome, is also available for minimally invasive techniques as a valid therapeutic approach in very select patients with localised lymph-nodal recurrence of gynaecological cancers (Gallotta et al., 2018).

Other Authors have presented their technique to treat para-aortic nodal metastasis in AOC (Uccella et al., 2020), with different laparoscopic access (traditionally, the laparoscopic view comes parallel to the main axis of the vascular structures).

According to our experience, the use of a 30-degree 3D-endoscope greatly helps in achieving the cranial landmarks of para-aortic dissection, holding the endoscope perpendicular to the vessels in the supraumbilical access. Choosing a more cranial laparoscopic access also helps in maintaining the same setting during the procedure, working both in the pelvis and abdomen. The leading and assisting surgeons do not have to move and re-set during the procedure, saving precious time. The role of the Anaesthetist is essential to give the high degree of Trendelenburg for a long time during the laparoscopic procedure. Together with the technical aspects of the procedure, we believe that the key-points to achieve full laparoscopic removal of disease are:

- proper selection of cases
- extensive experience of the surgeons
- deep knowledge of surgical anatomy of the retroperitoneum

In order to help the surgeon to tailor the best surgical approach to achieve complete tumour cytoreduction, several studies are focusing on the improvement of pre-operative work up, particularly the detection of suspicious lymph nodes with radiology (Gallotta et al., 2021) and carcinomatosis recognition, through integration of radiomic, machine learning, and ﬂuorescence- driven techniques (Nougaret et al., 2021).

The challenge of cancer surgery is to achieve radical treatment, striving to reduce loss of function and to preserve quality of life after surgery: as extensively reported for other complex gynaecological conditions (Ceccaroni et al., 2013), only the deep knowledge of surgical anatomy, particularly the knowledge of the retroperitoneum and its topographical complexity (Freytag et al., 2020) allows the surgeon to safely perform challenging procedures, including aortic resection of bulky nodes and cytoreduction of advanced gynaecological cancers.

## Conclusions

Repositioning the surgical field to perform para-aortic dissection can be a time-consuming procedure during laparoscopic debulking for ovarian cancer. According to our experience, laparoscopic removal of bulky para-aortic nodes with a 30-degree 3D-endoscope and a “top-bottom view” is feasible, safe and effective.

## Video scan (read QR)

https://vimeo.com/670629926/78b149621e

**Figure:**
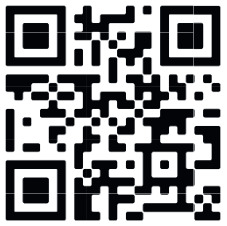


## References

[1] Ceccaroni M, Clarizia R, Roviglione G (2013). Neuroanatomy of the posterior parametrium and surgical considerations for a nerve-sparing approach in radical pelvic surgery. Surg Endosc.

[2] Fagotti A, Ferrandina G, Fanfani F (2006). A laparoscopy-based score to predict surgical outcome in patients with advanced ovarian carcinoma: a pilot study. Ann Surg Onc.

[3] Freytag D, Pape J, Dhanawat J (2020). Challenges Posed by Embryonic and Anatomical Factors in Systematic Lymphadenectomy for Endometrial Cancer.. J Clin Med.

[4] Gallotta V, Ghezzi F, Vizza E (2016). Laparoscopic Management of Ovarian Cancer Patients with Localized Carcinomatosis and Lymph Node Metastases: Results of a Retrospective Multi-institutional Series J Minim Invasive Gynecol.

[5] Gallotta V, Jeong SY, Conte C (2021). Minimally invasive surgical staging for early-stage ovarian cancer: A long-term follow up.. Eur J Surg Oncol.

[6] Gallotta V, Giudice MT, Conte C (2018). Minimally invasive salvage lymphadenectomy in gynecological cancer patients: A single institution series.. Eur J Surg Oncol.

[7] Gueli Alletti S, Capozzi VA, Rosati A (2019). Laparoscopy vs. laparotomy for advanced ovarian cancer: a systematic review of the literature. Minerva Med.

[8] Nougaret S, McCague C, Tibermacine H (2021). Radiomics and radiogenomics in ovarian cancer: a literature review. Abdominal Radiology.

[9] Querleu D, Planchamp F, Chiva L (2017). European Society of Gynaecological Oncology (ESGO) Guidelines for Ovarian Cancer Surger. International Journal of Gynecological Cancer.

[10] Uccella S, Zorzato PC, Forliti E (2020). Laparoscopic Excision of a 5-cm Retroaortic Relapse of Ovarian Cancer.. Journal of Minimally Invasive Gynecology.

